# Soybean yield in relation to distance from the Itaipu reservoir

**DOI:** 10.1007/s00484-015-1093-8

**Published:** 2015-11-03

**Authors:** Rogério Teixeira de Faria, Ruy Casão Junior, Simone Silmara Werner, Luiz Antônio Zanão Junior, Gerrit Hoogenboom

**Affiliations:** Univ. Estadual Paulista, Via de Acesso Prof. Paulo Donato Castellane s/n, Jaboticabal, SP 14884-900 Brazil; Fapeagro, Rua Paranaguá, 1672, Londrina, PR 86015-030 Brazil; Epagri, Rua João José Godinho, S/N, Lages, SC 88502970 Brazil; Iapar, BR 163, km 188, Santa Tereza do Oeste, PR 85825-000 Brazil; Washington State University, 24106 North Bunn Road, Prosser, WA 99350-8694 USA

**Keywords:** Water balance, Water stress, Yield components, Air temperature

## Abstract

Crops close to small water bodies may exhibit changes in yield if the water mass causes significant changes in the microclimate of areas near the reservoir shoreline. The scientific literature describes this effect as occurring gradually, with higher intensity in the sites near the shoreline and decreasing intensity with distance from the reservoir. Experiments with two soybean cultivars were conducted during four crop seasons to evaluate soybean yield in relation to distance from the Itaipu reservoir and determine the effect of air temperature and water availability on soybean crop yield. Fifteen experimental sites were distributed in three transects perpendicular to the Itaipu reservoir, covering an area at approximately 10 km from the shoreline. The yield gradient between the site closest to the reservoir and the sites farther away in each transect did not show a consistent trend, but varied as a function of distance, crop season, and cultivar. This finding indicates that the Itaipu reservoir does not affect the yield of soybean plants grown within approximately 10 km from the shoreline. In addition, the variation in yield among the experimental sites was not attributed to thermal conditions because the temperature was similar within transects. However, the crop water availability was responsible for higher differences in yield among the neighboring experimental sites related to water stress caused by spatial variability in rainfall, especially during the soybean reproductive period in January and February.

**Electronic supplementary material:**

The online version of this article (doi:10.1007/s00484-015-1093-8) contains supplementary material, which is available to authorized users.

## Introduction

The crop yield at sites neighboring small water bodies, such as artificial reservoirs, may be affected by microclimatic alterations caused by the presence of the water mass (Klaić and Kvakić 2014). The Itaipu reservoir was artificially formed in 1982 and has a total area of 1350 km^2^, storage volume of 29 × 10^9^ m^3^, length of 170 km, and mean width of 7 km. The area 10 km around the lake border is 147,676 ha, in which 96,967 ha are occupied by agricultural crops, mostly soybean cultivated during the Brazilian spring-summer in succession with the winter corn crop. The reservoir is located in the Western region in Paraná State, which is the largest soybean producer because of the fertile soils, suitable climate, excellent cooperative organization, and high technological level among the farmers.

Reports in the scientific literature on large water bodies (Sanderson 2004 and Awange and Osienala, 2006) or even small artificially created reservoirs (Klaić and Kvakić 2014) indicated that these water bodies can affect the microclimate of adjacent areas, thus altering the weather conditions throughout the day. The different thermodynamic characteristics between land and water results in a thermal gradient that promotes circulation of air masses by lake breezes, which mainly alter air temperature and humidity (Crosman and Horel 2010) and affect crop growth at the neighboring sites (Sanderson 2004).

Previous analyses performed with a mesoscale climate simulation model called NH-TVM indicated that the Itaipu reservoir is capable of inducing and maintaining lake breeze circulation (Stivari et al. 2003) and causing reduced amplitudes of the air temperature up to 5 km away from the shoreline (Stivari et al. 2005). In another study that tested if the spatial behavior of the air temperature is affected by distance from the Itaipu reservoir, Wagner-Riddle et al. (2015) analyzed a data series of air temperature and wind speed and direction measured in seven transects in a 10-km-wide area for 3 years. The results obtained by Wagner-Riddle et al. (2015) are inconsistent with the conclusions of Stivari et al. (2003, 2005) because differences in temperature along with distance from the reservoir were not observed, and these authors also observed a weak or nonexistent lake breeze that has a low potential to alter the daily thermal amplitude. They suggested that intensive latent heat flux associated with soybean evapotranspiration would induce lower sensible heat fluxes than required for strong lake breeze development. In addition, Wagner-Riddle et al. (2015) argued also that the small width (∼7 km) of the water body, together with the presence of the 200-m vegetative strip with a tall canopy of large aerodynamic roughness length, would also be detrimental for lake breeze development as the frictional drag works to diminish the development of horizontal pressure gradients associated with lake breezes.

Although there is a vast literature on the effects of large water bodies on neighboring microclimate, any previous published work demonstrating such effect on field crops is not known. Therefore, it is necessary to clarify if crop yield, particularly of soybean, because it is the species of highest economic importance within the region, is affected by alleged microclimatic alterations caused by the Itaipu reservoir’s formation.

Soybean grain yield is a result of genetics, climatogy, soil characteristics, cultural practices, and stress applied to the plants during the growth cycle. The first three factors define the crop yield potential, whereas the different stresses reduce the level of production relative to the defined potential (Cassman et al. 2011). In the Itaipu reservoir, eventual microclimatic differences between the sites surrounding the reservoir that would affect yield should primarily be related to air temperature and water availability caused by variability in rainfall.

Changes in temperature within a crop’s sensitivity range impact the physiological processes and yield (Board and Kahlon 2011). Variations in air temperature mainly affect the duration of phenological stages, carbon balance (photosynthesis and respiration), and growth, abortion, and senescence of plant organs (Boote et al. 2010). For soybean, increased temperature affects grain yield by shortening the phenological stages (Seddigh and Joliff 1984), especially the duration of seed development and grain filling stages (Boote 2011), resulting in smaller seeds and lower yields (Egli and Wardlaw 1980). Temperatures above 40 °C reduce photosynthetic capacity and canopy development, and temperatures below 15 °C can negatively affect vegetative development, flowering, and yield components (Board and Kahlon 2011; Embrapa 2011). Diurnal temperature variations between 26 and 36 °C do not cause significant alterations in photosynthesis and are not considered an important factor in defining soybean grain yield (Campbell et al. 1990; Gibson and Mullen 1996; Boote 2011). In the sites neighboring the Itaipu reservoir, the mean and maximum temperatures during the soybean reproductive phase in January and February are between 25 and 27 °C and 32 and 34 °C, respectively; thus, they are within the temperature range considered to produce a low response to photosynthesis. Growth respiration (growth conversion efficiency) is unaffected by temperatures below the extreme values (Penning de Vries et al. 1974); however, maintenance respiration increases with increasing temperature (Mccree 1974) and consumes assimilates, which reduces assimilate availability for biomass accumulation and decreases soybean grain yield (Board and Kahlon 2011). Because soybean is a C3 plant, it is less sensitive to increased respiration because of increased nocturnal temperatures (Peters et al. 1971).

Water availability is considered a limiting factor for crop yield. Physiologically, water stress affects photosynthesis by reducing gas exchanges related to stomatal closure, thus reducing the growth rate of plant organs and development of seeds and causing abortion and accelerated senescence of vegetative and reproductive structures (Boote et al. 2010). The effect of water stress on soybean production depends on the intensity of the stress and period in which it occurs. Water deficits that occur during soybean’s vegetative stage reduce the leaf area, dry weight, diameter, and length of the main branch and number and length of the internodes (Board and Kahlon 2011). Internodes that begin development during stress periods exhibit reduced length (Desclaux et al 2000). Water deficits that coincide with the flowering and pod set stages lead to increased abortion of flowers and pods. The final number of pods depends on climactic conditions and plant vigor during the flowering period. However, water deficits become critical for soybeans during the grain filling stage because such stress reduces the seed size and weight, number of grains per plant, and grain composition (Embrapa 2011; Yee-Shan Ku et al 2013). The grain unit weight is most frequently used parameter to show this effect.

The Itaipu reservoir’s modification of the local microclimate, and consequent alterations to the yield of adjacent soybean plants, are gradual effects that exhibit greater intensity at sites near the shoreline and decrease with increasing distance from the reservoir. To test this hypothesis, this study aimed to evaluate soybean yield in relation to distance from the reservoir and determine the effect of water availability and air temperature on yield, phenology, and yield components of the soybean crop.

## Material and methods

**Fig. 1:**
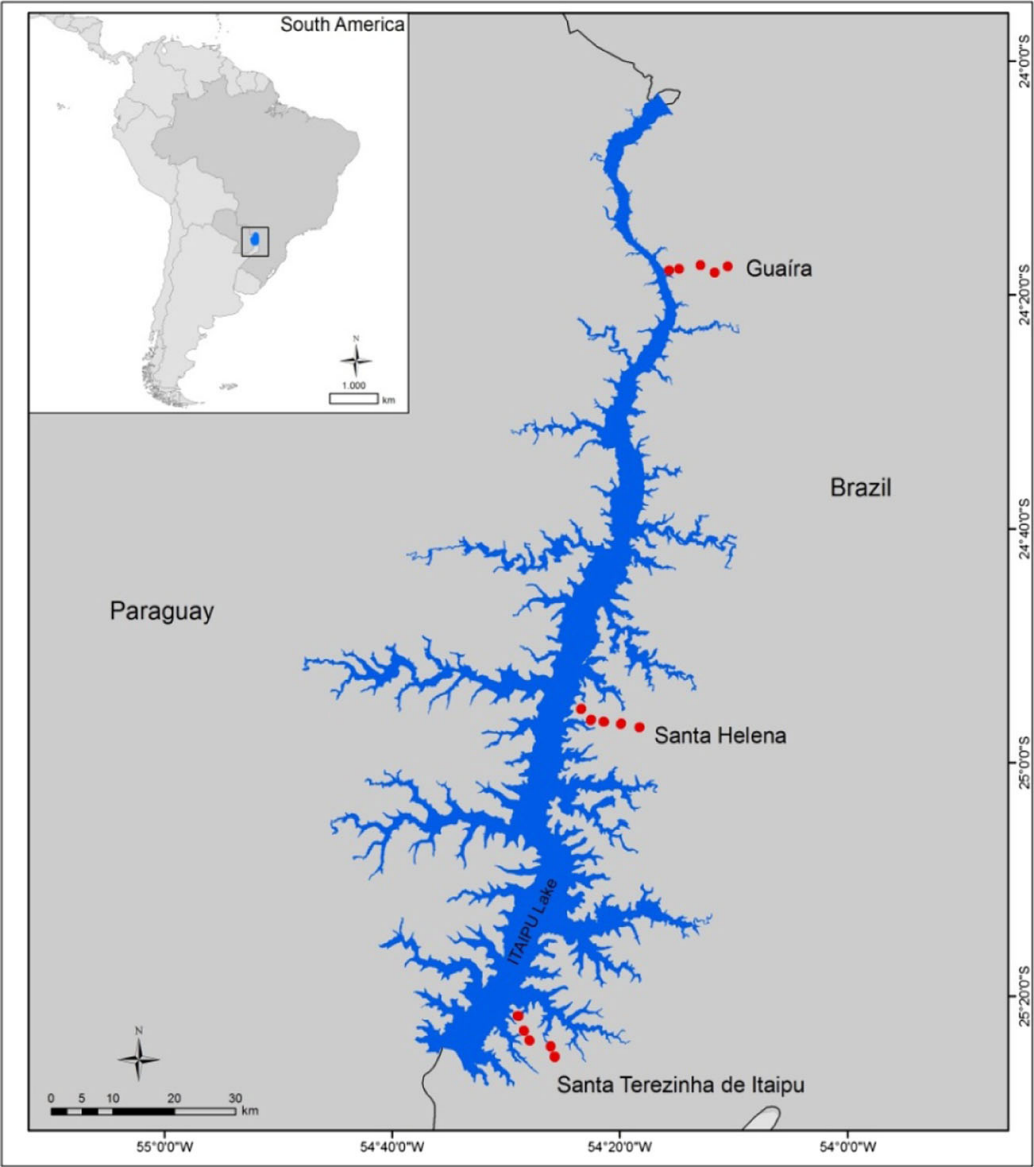
Location of the experimental sites in three transects perpendicular to the Itaipu reservoir

The experiments were conducted in three transects that were used to obtain the weather data for the analysis by Wagner-Riddle et al. (2015), during four consecutive crop seasons (2010/2011 to 2013/2014) in the locations of Santa Terezinha de Itaipu (STI), Santa Helena (SHE), and Guaíra (GUA), which are in Paraná State, Brazil (Fig. 1). The local climate is humid subtropical (Cfa) according to the Köppen classification, and the area has a mean annual temperature of 21.5 °C, annual reference evapotranspiration of 1100 to 1200 mm, and annual rainfall between 1600 and 1800 mm (Iapar 1994). During the soybean crop cycle, from October to February, the mean cumulative rainfall varies from 700 to 825 mm and reference evapotranspiration varies from 595 to 680 mm, with 400- to 475-mm rainfall and 365- to 420-mm evapotranspiration occurring from December to February.

Five experiments were conducted in each of the three locations in 1-ha areas arranged in transects perpendicular to the Itaipu reservoir (Fig. 1). The experimental sites (1, 2, 3, 4, and 5) were selected according to their distance from the reservoir’s body, which ranged from 0.3 to 1 km, 1 to 3 km, 3 to 5.5 km, 5.5 to 8.5 km, and 8.5 to 12 km, respectively (Table 1). Sites that had altitudes lower than 325 m, mean slopes lower than 8 %, western exposure and soils classified as Red Latosol with very clayey texture, and were farmed under no-tillage were selected for the experiment (Table 1).

**Table 1:** Characteristics of the experimental sites in each transect

Code	Soil	Altitude (m)	Distance from reservoir (km)	Latitude (S)	Longitude (W)
Santa Terezinha de Itaipu (STI)					
STI1	LVd	238	1.04	25° 21′ 39.7″	54° 28′ 52.3″
STI2	LVdf	268	3.42	25° 22′ 57.3″	54° 28′ 22.5″
STI3	LVd	264	5.01	25° 23′ 44.2″	54° 27′ 55.7″
STI4	LVdf	246	7.78	25° 24′ 14.2″	54° 26′ 02.8″
STI5	LVdf	275	9.24	25° 25′ 07.1″	54° 25′ 42.9″
Santa Helena (SHE)					
SHE1	LVdf	250	1.20	24° 55′ 21.6″	54° 23′ 24.5″
SHE2	LVdf	260	3.87	24° 56′ 23.8″	54° 22′ 20.8″
SHE3	LVdf	284	5.28	24° 56′ 37.0″	54° 21′ 30.1″
SHE4	LVef	239	7.81	24° 56′ 36.5″	54° 19′ 51.9″
SHE5	LVef	296	10.59	24° 56′ 56.6″	54° 18′ 14.8″
Guaíra (GUA)					
GUA1	LVef	275	0.74	24° 17′ 54.1″	54° 15′ 40.9″
GUA2	LVef	295	2.14	24° 17 ′43.2″	54° 14′ 49.0″
GUA3	LVef	287	5.41	24° 17′ 24.7″	54° 12′ 56.4″
GUA4	LVef	321	7.47	24° 18′ 04.8″	54° 11′ 41.2″
GUA5	LVef	302	9.38	24° 17′ 34.4″	54° 10′ 31.3″

*LVd* dystrophic Red Latosol (Haplustox), *LVdf* dystroferric Red Latosol (Rhodic Ustox), *LVef* eutroferric Red Latosol (Haplustox) (Embrapa 2013)

**Fig. 2:**
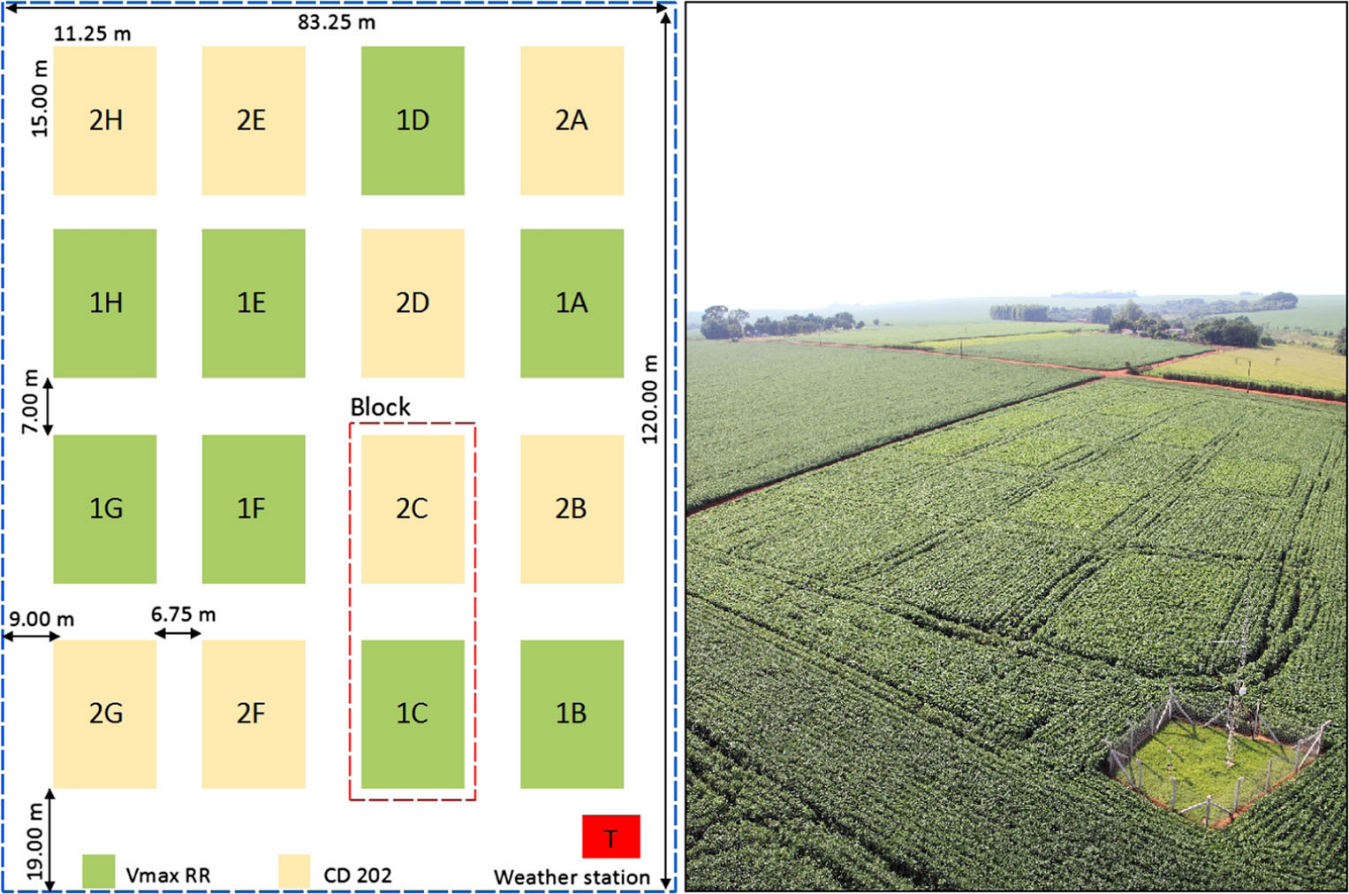
Schematic representation of an experimental site and plot layout

The experiment was conducted in a randomized block design with eight replicates (Fig. 2). Soybean cultivars with determinate and indeterminate growth were sowed in each block, and there were 16 plots in each experimental site. Each plot contained 25 14-m-long rows spaced 0.45 m apart.

The two evaluated soybean cultivars, CD202 and Vmax RR, were selected because they were the most frequently cultivated in the region when the experiments were initiated. These cultivars have semi-early cycles (VE-R7 of approximately 120 days), Vmax RR is transgenic and has indeterminate growth, and CD202 is non-transgenic and has determinate growth. However, as of the 2012/2013 crop season, the CD202 cultivar was no longer available on the market and was replaced by CD202RR, which has a transgenic gene.

The soil fertility of the sites was corrected and standardized during the crop season preceding the establishment of the experiment. The base saturation and nutrient levels were corrected for values above the critical level for each nutrient according to levels established by Embrapa (2010). To produce a uniform and standard straw cover, black oat (*Avena strigosa* Schreb) cultivar Iapar-61 was grown during the Brazilian winter prior to sowing soybean crops at all of the sites.

The experiments were sowed during October in every crop season, and the five experimental sites of each transect were sown with soybean on a single day in each crop season. The plant population ranged from 300,000 to 350,000 plants ha^−1^ for the Vmax RR cultivar and from 220,000 to 280,000 plants ha^−1^ for the CD202 cultivar. The seeds were previously treated with fungicide and insecticide and inoculated with *Bradyrhizobium japonicum* strains at sowing. Additional crop management practices to control diseases, pests, and weeds were performed following recommendations by Embrapa (2010, 2011).

A chemical analysis of the soil from each experimental plot was performed annually at 0–10- and 10–20-cm soil layers to determine the appropriate amounts of fertilizer for the nutritional requirements of the soybean plants (Embrapa 2010, 2011). Grain size distribution and water retention curves were also determined during the first crop season using data provided by Richards chamber.

Soybean yield and grain moisture content were determined in the plants of the 12 central rows of the plot, which were harvested with a plot harvester (Classic-Wintersteiger, Ried, Austria). Grain moisture content was corrected to 13 %, and the impurities (soil particles, straw and grain of other species) and harvest losses were quantified. The initial plant stand was evaluated immediately after emergence, and the yield components were determined in eight sub-samples of 1-m plant rows sampled randomly from the border of the harvested area of the plot.

An automatic weather station (Campbell Scientific, Ltd, Logan, UT, USA) was installed at each experimental site within a fenced grassy area. The station contained sensors for measuring global radiation, air temperature, relative humidity, wind speed/direction, and rainfall (Fig. 2).

Water availability during the crop cycles in each crop season was characterized by three indices: rainfall, water deficit, and water stress. These indices were accumulated during the crop cycles or phenological stages in each cycle according to the scale by Fehr and Caviness (1977). The water deficit index was calculated by determining the differences between the rainfall and reference evapotranspiration (ETo) amounts during the crop cycles or phenological stages. Reference evapotranspiration was estimated by the standard FAO-56 (Allen et al. 1998) using daily data obtained from the weather stations. The water stress index was estimated by the Cropping System Model (CSM)-CROPGRO-Soybean (Hoogenboom et al 2012) by simulations performed for each crop cycle using data on the local weather and crop management and parameterized genetic coefficients of the cultivars and soil characteristics for each experimental site. The daily water stress (Daily stress) varied from zero (no stress) to one (maximum stress), as a calculation based on a comparison of potential root uptake (TRWU) and atmospheric potential demand (EP), according to:

1 $$ \begin{array}{c}\hfill \kern1.5em \mathrm{If}\ \mathrm{TRWU}\ge 1.5\ \mathrm{E}\mathrm{P},\ \mathrm{then}\ \mathrm{Daily}\ \mathrm{stress}=0\kern4em \hfill \\ {}\hfill \mathrm{If}\kern0.5em \mathrm{TRWU}<1.5\ \mathrm{E}\mathrm{P},\kern0.5em \mathrm{then}\kern0.5em \mathrm{Daily}\kern0.5em \mathrm{Stress}=1-\frac{\mathrm{TRWU}}{1.5\ \mathrm{E}\mathrm{P}}\hfill \end{array} $$

in which TRWU is a function of root length density, rooting depth, root distribution, and the actual soil water content for the layers where roots are present, and EP is calculated from ETo and leaf area index (White and Hoogenboom, 2010). Cumulative water stress indices (Stress) were calculated for the crop cycles or phenological stages as the sum of the daily values of stress index to quantify the severity of drought, similarly to the procedure described by Soler et al. (2013). The Genetic coefficients of Vmax and CD202 cultivars were estimated using field data from experiments conducted in 2010/11 and 2011/12. A Bayesian parameter estimation procedure (the Generalized Likelihood Uncertainty Estimation, or GLUE) (Jones et al, 2010) was used to select cultivar parameters that gave the least deviation of simulated to observed phenological and growth data. The capability of the (CSM)-CROPGRO-Soybean to estimate observed yield was evaluated using observed data from the 2012/13 and 2013/14 crop seasons. The comparison of the simulations to observed dates of phenological stages (R1, R5, and R8), as well as final biomass, grain yield, and yield components were in good agreement under different weather conditions. An inverse relationship between the simulated cumulative water stress index and observed soybean yield was found to be closely correlated, indicating the ability of the model to predict yield decrease due to water stress.

The mixed model method was used in the soybean yield data analysis for each transect and crop season, and the following factors were considered: block nested in each experimental site, cultivar (with two levels), experimental site or distance (with five levels), and interactions between the factors cultivar and distance. The following four structures were tested to determine the matrix of variances and covariances: unstructured variance components, heterogeneous variance components, and compound symmetry. The restricted maximum likelihood (REML) method (Harville 1977) and SAS software (SAS 2008) were employed for the estimations. In non-nested models, the likelihood ratio test or Akaike information criterion were used to perform selections. The *F* test and *Satterthwaite* method were employed to approximate the degrees of freedom (Sattherthwaite 1946) and test for significance among the effects in the fixed portion of the model. All of the analyses were performed according to a 5 % significance level.

Contrast tests were performed when a significant effect was observed among the experimental sites (distances) or interactions occurred between the cultivar and experimental sites. Six contrasts of interest were defined, which comparisons prioritized between experimental sites that were closer to and farther from the Itaipu reservoir:

$$ \begin{array}{ll}{\mathrm{H}}_{01:}\ {\mu}_1\hbox{--} {\mu}_2=0\hfill & \left(\mathrm{Distance}\kern0.5em 1\kern0.5em \mathrm{v}\mathrm{s}\kern0.5em \mathrm{Distance}\ 2\right)\hfill \\ {}{\mathrm{H}}_{02:}\ {\mu}_1\hbox{--} {\mu}_3=0\hfill & \left(\mathrm{Distance}\ 1\ \mathrm{v}\mathrm{s}\kern0.5em \mathrm{Distance}\ 3\right)\hfill \\ {}{\mathrm{H}}_{03:}\ {\mu}_1\hbox{--} {\mu}_4=0\hfill & \left(\mathrm{Distance}\ 1\ \mathrm{v}\mathrm{s}\kern0.5em \mathrm{Distance}\ 4\right)\hfill \\ {}{\mathrm{H}}_{04:}\ {\mu}_1\hbox{--} {\mu}_5=0\hfill & \left(\mathrm{Distance}\ 1\kern0.5em \mathrm{v}\mathrm{s}\kern0.5em \mathrm{Distance}\ 5\right)\hfill \\ {}{\mathrm{H}}_{05:}\ 4\ {\mu}_1\hbox{--}\ {\mu}_2\hbox{--}\ {\mu}_3\hbox{--}\ {\mu}_4\hbox{--}\ {\mu}_5=0\hfill & \left(\mathrm{Distance}\ 1\ \mathrm{v}\mathrm{s}\kern0.5em \mathrm{Distance}\ 2 + \mathrm{Distance}\ 3 + \mathrm{Distance}\ 4 + \mathrm{Distance}\ 5\right)\hfill \\ {}{\mathrm{H}}_{06:}\ 3\ {\mu}_1 + 3\ {\mu}_2\hbox{--}\ 2\ {\mu}_3\hbox{--}\ 2\ {\mu}_4\hbox{--}\ 2\ {\mu}_5 = 0\hfill & \left(\mathrm{Distance}\ 1 + \kern0.5em \mathrm{Distance}\ 2\ \mathrm{v}\mathrm{s}\kern0.5em \mathrm{Distance}\ 3 + \mathrm{Distance}\ 4 + \mathrm{Distance}\ 5\right)\hfill \end{array} $$

In this analysis, a contrast was a linear combination of yield means whose coefficient values add up to zero, allowing comparison of different treatments. For instance, contrast 1 allows to test if there is a statistically significant difference between the observed mean yield at the sites in the first and the second distance, while contrast 5 compares the observed mean yield at the site closest to the Itaipu reservoir and mean yield at the other sites farther away from the reservoir. Contrast 6 can be used to analyze the same effect, although it compares the mean yield between the two closest sites with the mean yield of the three farthest sites from the reservoir.

## Results and discussion

**Fig. 3:**
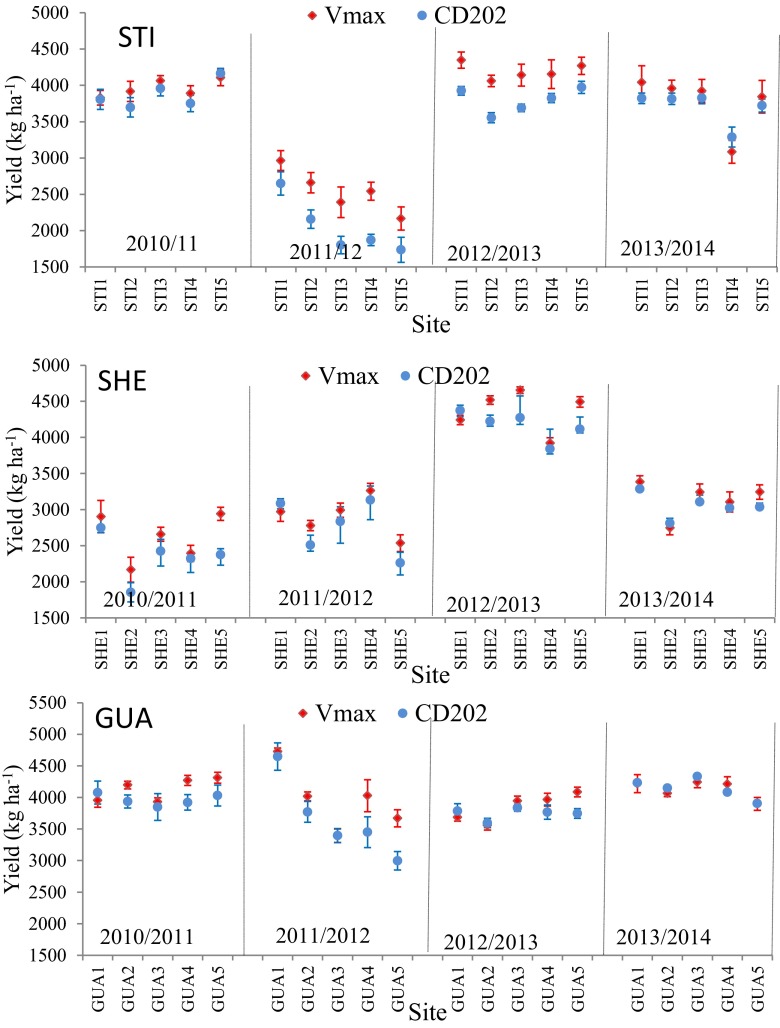
Mean soybean yield of the two soybean cultivars in each transect according to the crop season and experimental sites at five distances from the Itaipu reservoir. *Error bars* indicate 95% confidence interval

Soybean yield varied according to transect, crop season, cultivar, and distance from the reservoir (Fig. 3), with stable yields throughout the crop seasons and higher means observed among the transects in GUA (3947 kg ha^−1^), intermediate mean yields observed in STI (3485 kg ha^−1^), and lower mean yields observed in SHE (3214 kg ha^−1^). The Vmax cultivar exhibited higher mean yields (3647 kg ha^−1^) compared with CD202 (3450 kg ha^−1^), although the mean yield in relation to distance from the reservoir in the transects did not exhibit a clear trend and varied according to distance, crop season, and cultivar.

In more than 70 % of the contrasts, differences in soybean yield between site 1 or site 2 and sites farther from the reservoir were lower than 10 % of the mean yield of the experiments, i.e., 350 kg ha^−1^ (Tables 2 and 3). The low values of the contrasts with statistical significance reflected excellent experimental control as indicated by coefficients of variation lower than 10 %.

**Table 2:** Estimated values of the contrasts (kg ha^−1^) according to transect and crop season in the cases where the cultivar × distance interaction was significant

Contrast	Transect								
	STI			SHE			GUA		
	2011/2012	2012/2013	2013/2014	2010/2011	2012/2013	2013/2014	2010/2011	2011/2012	2012/2013
Vmax									
Distance 1 vs Distance 2	**305.18**	**287.29**	83.70 ns	**735.93**	−**281.99**	**638.27**	−**243.77**	**711.39**	**124.94**
Distance 1 vs Distance 3	**574.86**	**206.01**	118.12 ns	**244.96**	−**407.27**	**140.87**	23.34 ns	**1335.59**	−**259.73**
Distance 1 vs Distance 4	**422.60**	**195.01**	**955.96**	**509.44**	177.43 ns	**277.69**	−**317.11**	**700.06**	−**280.63**
Distance 1 vs Distance 5	**798.33**	78.19 ns	**197.34**	−38.46 ns	−**523.42**	**140.63**	−**359.16**	**1058.20**	−**401.29**
Distance 1 vs Distance 2 + Distance 3 + Distance 4 + Distance 5	**525.24**	**191.62**	**338.78**	**362.97**	−**258.81**	**299.36**	−**224.18**	**951.31**	−**204.18**
Distance 1 + Distance 2 vs Distance 3 + Distance 4 + Distance 5	**446.01**	16.09 ns	**381.96**	−**129.32**	−110.09 ns	−**132.74**	−95.76 ns	**675.59**	−**376.35**
CD202									
Distance 1 vs Distance 2	**491.78**	**370.58**	7.29 ns	**893.85**	**105.05**	**473.86**	141.05 ns	**876.58**	**190.53**
Distance 1 vs Distance 3	**849.04**	**236.01**	−4.97 ns	**323.35**	141.68 ns	**180.36**	**228.55**	**1249.87**	−50.44 ns
Distance 1 vs Distance 4	**779.43**	**100.33**	**534.63**	**425.22**	**379.51**	**264.36**	154.59 ns	**1197.48**	17.82 ns
Distance 1 vs Distance 5	**914.52**	−46.31 ns	99.80 ns	**372.61**	−17.23 ns	**251.43**	45.61 ns	**1650.23**	38.73 ns
Distance 1 vs Distance 2 + Distance 3 + Distance 4 + Distance 5	**758.69**	**165.15**	**159.19**	**503.76**	**152.25**	**292.50**	142.45 ns	**1243.54**	49.16 ns
Distance 1 + Distance 2 vs Distance 3 + Distance 4 + Distance 5	**601.77**	−**88.61**	**206.17**	−73.20 ns	115.46 ns	−4.88 ns	72.39 ns	**927.57**	−**93.23**

Values in bold indicate statistically significant *F* test values

*ns* not significant (*p* value ≤ 0.05)

**Table 3:** Estimated values of the contrasts (kg ha^−1^) according to transect and crop season in the cases in which the cultivar × distance interaction was not significant

Contrast/transect	STI	SHE	GUA
	2010/2011	2011/2012	2013/2014
Distance 1 vs Distance 2	10.44 ns	**382.94**	**120.06**
Distance 1vs Distance 3	−**192.60**	114.34 ns	−62.60 ns
Distance 1vs Distance 4	−3.17 ns	−**170.37**	77.64 ns
Distance 1 vs Distance 5	−**317.31**	**629.69**	**323.75**
Distance 1 vs Distance 2 + Distance 3 + Distance 4 + Distance 5	−**125.66**	**239.15**	**114.71**
Distance 1 + Distance 2 vs Distance 3 + Distance 4 + Distance 5	−**176.25**	−0.25 ns	52.90 ns

Values in bold indicate statistically significant *F* test values

*ns* not significant (*p* value ≤ 0.05)

Among the four crop seasons, the cultivar × distance interaction was not significant in one crop season in SHE, one crop season in STI, and one crop season in GUA (Table 3). The significance of the cultivar × distance interaction demonstrates that the effect of the cultivar is dependent on the site where it is cultivated (distance) because at certain sites, the yield of Vmax RR was higher than the yield of CD202, whereas the opposite occurred in other sites. Thus, a consistent pattern was not observed for cultivar yield in relation to distance, which may be explained by the physiological characteristics of the cultivars in response to the variations in climatic conditions at the sites in the different crop seasons and transects.

The values of the contrasts estimated during the different crop seasons did not show consistent trends, which is indicated by the changing sign in the estimates according to crop season and transect (Tables 2 and 3). For the cases in which the interaction was significant for Vmax RR (Table 2), contrast 5 exhibited a higher observed mean yield at distance 1 than at the farther sites in six of the nine cases. Considering the same contrast for CD202, a higher yield was also observed at distance 1 compared with the yield of sites farther away in seven of the nine cases, and the differences were not significant in the other two cases. In the case of contrast 6 for Vmax RR (Table 2), the observed mean yield at distance 1 + distance 2 was higher than the observed mean yield at the sites farther away in three of the nine cases, differences were not significant in three cases, and yield was lower than the mean yield of the farthest sites in three cases. Considering the same contrast for CD202, the observed mean yield at distance 1 + distance 2 was higher than the mean yield of the farther sites in three of the nine cases, differences were not significant in four cases, and yield was lower in two cases.

For the cases in which there was no interaction between cultivars and distance (Table 3), the contrasts ranged from −317.31 to 10.44 kg ha^−1^ in STI (2010/2011), from −170.37 to 629.69 kg ha^−1^ in SHE (2011/2012), and from −62.60 to 323.75 kg ha^−1^ in GUA (2013/2014); thus, 6 of 18 cases presented higher contrasts at the sites closer to the reservoir, 7 cases were not significant, and 5 presented yields that were lower than the mean yield of the farther sites.

The statistically significant differences revealed by the contrasts may be attributed to the prevalent production factors at the different experimental sites during the study. The spatial variability of the soil attributes was minimized by selecting experimental sites that had the same soil types and uniform soil fertility and applying the same crop management practices to all sites; therefore, the variation in soybean yield among the sites of the transects (Fig. 3) was most likely caused by the weather conditions of each experimental site.

**Fig. 4:**
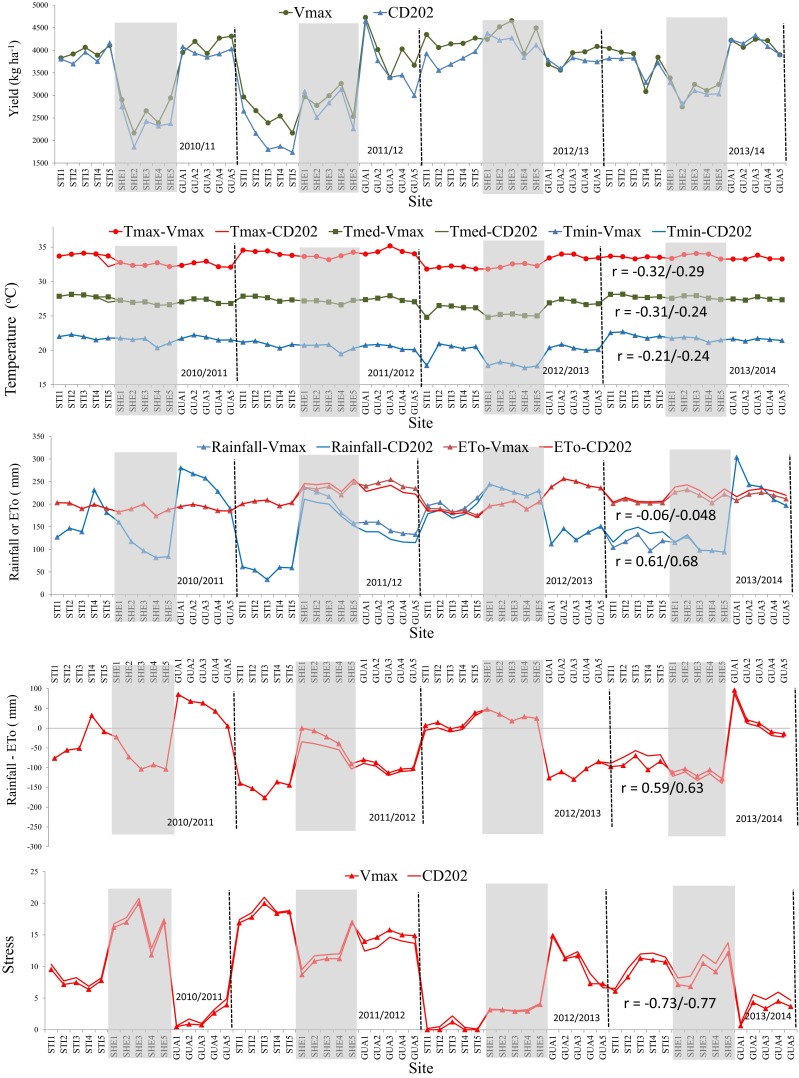
Soybean yield, temperature (*Tmax*, *Tmed*, and *Tmin*), evapotranspiration (*ETo*), rainfall (*R*), water deficit (*R-ETo*), and water stress (*Stress*) during the grain filling stage (R5–R7), according to experimental site and crop season. The figure shows the correlation coeffient (*r*) between those variables and yields of the Vmax RR (*first value*) and CD202 (*second value*) cultivars

The effect of climatic factors on soybean yield is shown in Fig. 4, which presents the correlation between the mean yields of the cultivars and mean values of the weather variables during the grain filling stage (R5–R7) in the four analyzed crop seasons. A low correlation was observed among the maximum, median, and minimum temperatures and reference evapotranspiration with grain yield (*r* < 0.32). The same analysis revealed higher correlations among the yield of the Vmax RR and CD202 cultivars and rainfall (*r* = 0.61 and 0.68, respectively), water deficit (*r* = 0.59 and 0.63, respectively), and water stress indices (*r* = 0.73 and 0.77, respectively). Thus, the elements related to thermal factors explained less than 10 % of the variations in yield (*R*^2^ < 0.10), whereas those related to water factors explained 34 to 59 % of the variation in yield (0.34 ≥ *R*^2^ ≥ 0.59).

The low effect of air temperature on soybean yield can be explained by the similarity between the mean temperatures during the crop cycles and normal temperatures in the region, especially during the reproductive period in January and February. The normal temperatures in the areas surrounding of the Itaipu reservoir are between 25 and 27 °C (Iapar 1994), which is considered ideal for soybean growth (Boote 2011). The low correlation is also due to the fact that temperature variations are very low from year to year, from site to site, and in the transects. The results presented in Fig. 4 show that differences in the mean maximum and minimum temperatures among the sites within the same transect were lower than 1 °C in almost all of the crop cycles. Analyzing the same database, Wagner-Riddle et al. (2015) found that there was no difference in the air temperature with increasing distance from the shoreline of the Itaipu reservoir.

**Fig. 5:**
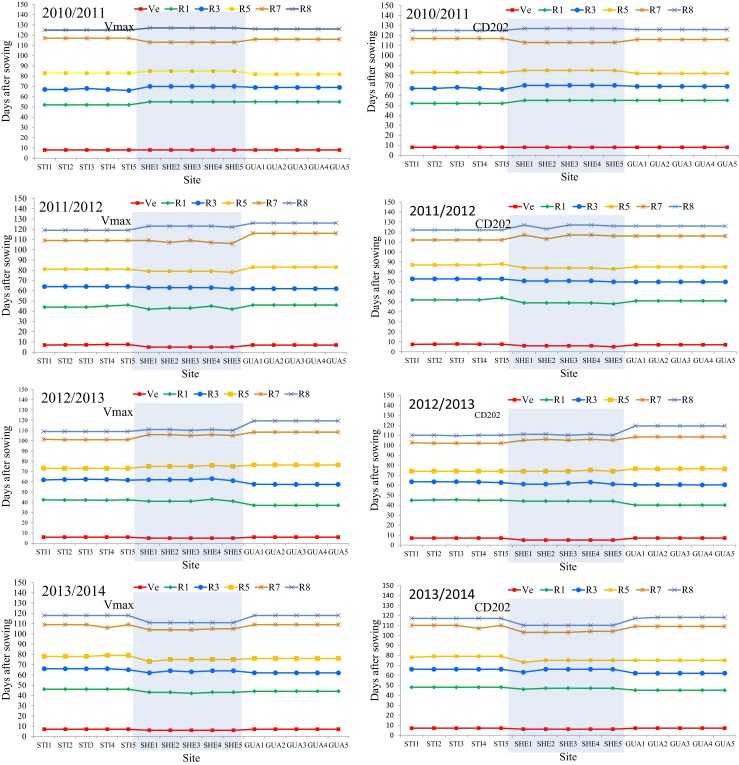
Dates of occurrence of the different phenological stages for the Vmax RR and CD202 cultivars according to experimental site, transect, and crop season

Because there were no differences in temperature throughout the transects, the soybean phenological stages occurred on the same approximate date for the two cultivars (Fig. 5). Based on the results of the correlation analysis between soybean yield and climatic variables (Fig. 4) and similarities in the duration of phenological stages among sites in the same transect (Fig. 5), the thermal conditions measured during the experimental period did not affect the soybean phenology and do not explain the observed variation in yield among the sites in the transects.

As the predominant wind direction in the region of the Itaipu reservoir is from the east and northeast, one should presume this effect could be a reason for the lack of influence of the lake on the temperature. A large lake in an arid environment can influence the humidity in the surroundings, especially on the side opposite to the prevailing wind direction due to temperature gradients, humidity, and pressure that may form between the surface of the water and the land. In semi-arid conditions, there are reports on breeze effects caused by large land-water contrast (Klaić and Kvakić 2014). In small lakes (width less than 50 km), conceptual evaluations about breeze formation have suggested that the main factor affecting the intensity of breeze is the magnitude of the sensible heat flux in the surrounding areas, which will be responsible for the formation of thermal gradients (SEGAL et al., 1997). However, in small lakes located in regions in which evapotranspiration is equivalent to rainfall, like in the region of the Itaipu reservoir, high evaporative flow and low sensible heat flux cause this effect to be reduced or nonexistent due to the low contrast land-water (Segal et al., 1997; Wagner-Riddle et al., 2015). In the absence of such a contrast, the potential of mesoscale wind to affect the surrounding farmland of the Itaipu reservoir is very low, even if the prevailing wind direction was the opposite, i.e., from west and southeast.

The high correlations between yield and variables related to water availability (Fig. 4) may be attributed to the high spatial variability of rainfall during the crop cycle, which is characteristic of the summer period in tropical and subtropical regions (Kim and Alexander 2013). According to Andresen et al. (2001), low precipitation and moisture stress were the major limitations to long-term regional crop yields of soybean, maize, and alfalfa in the Great Lakes region in North America. Wang et al. (2003) suggested that the variability in rainfall during the growth cycle or between crop seasons was more important in defining soybean yield than spatial variability. In the present study, this phenomenon unevenly affected the water availability for the crops at the experimental sites and caused differences in yield.

**Fig. 6:**
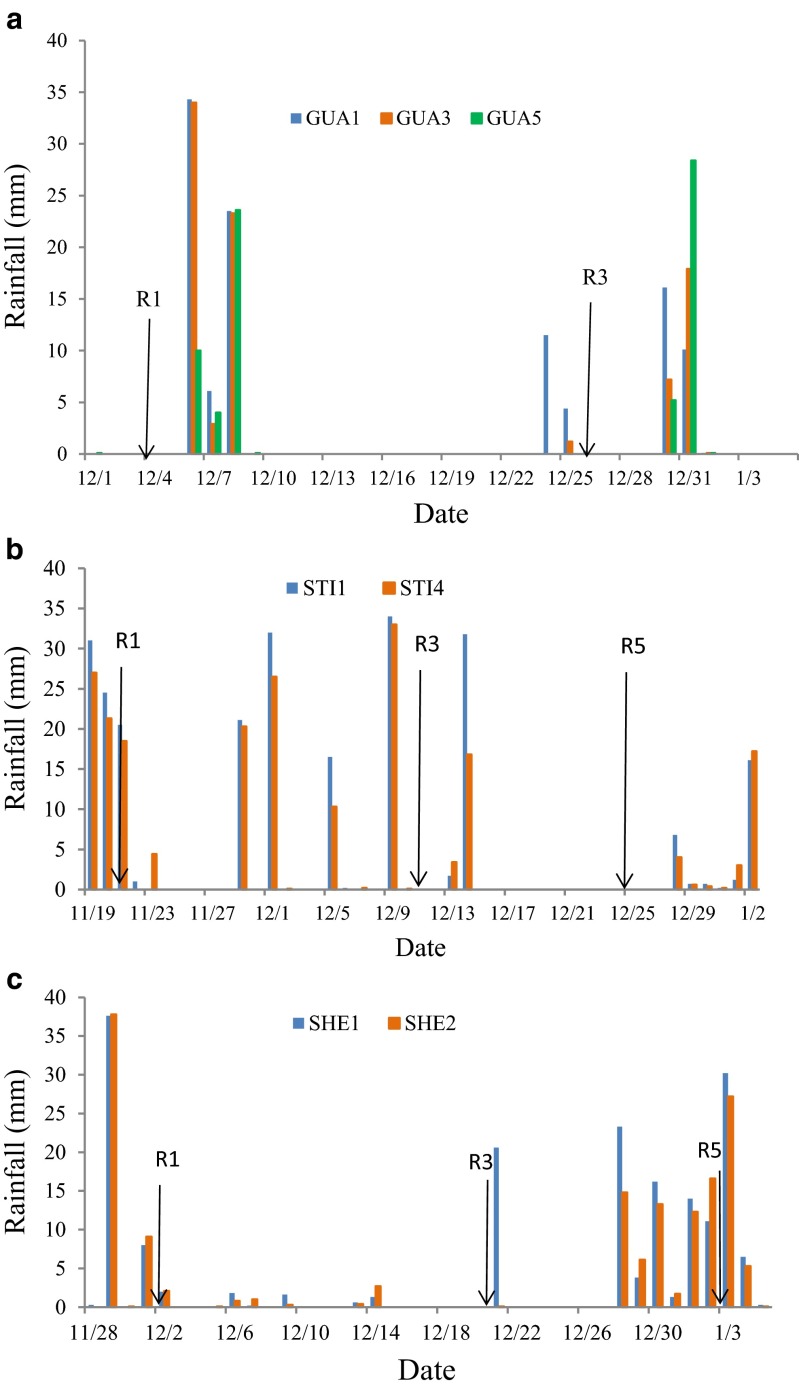
Rainfall during the flowering-pod set stage of the Vmax RR and CD202 cultivars during the 2011/12 crop season in Guaíra (*GUA1*, *GUA3*, and *GUA5*) (**a**) and 2013/14 crop season in Santa Terezinha de Itaipu (*STI1* and *STI4*) (**b**), and Santa Helena (*SHE1* and *SHE2*) (**c**)

Evidence that the water stress was responsible for the highest differences in yield among sites in the same transect, which is indicated by the contrasts in Tables 2 and 3, can be demonstrated by an analysis of yield and biometric data observed in relation to the water conditions of the transects. Thus, in the GUA transect during the 2011/2012 crop season, the GUA1 site exhibited higher mean yields than the GUA3 and GUA5 sites, with the contrast values for distance 1 vs distance 3 and distance 1 vs distance 5 ranging from 1336 to 1058 kg ha^−1^ for Vmax RR and 1250 to 1650 kg ha^−1^ for CD202 (Table 2). In this transect, the yields estimated by the product of the yield components were similar to those observed at the experimental sites (Table 4). The Vmax RR cultivar exhibited 39 % lower yield at the GUA3 site compared with the GUA1 site primarily because of a 17 % decrease in the number of pods per plant; however, the grain unit weight and plant population remained stable at both sites. Similarly, the Vmax RR cultivar exhibited a 55 % reduction in yield at the GUA5 site compared with the GUA1 site because of a 47 % reduction in the number of pods per plant. The reduced number of pods per plant at sites GUA3 and GUA5 was caused by water stress during the flowering and pod and grain formation stages (Embrapa 2011; Yee-Shan Ku et al 2013). A decrease in rainfall occurred during the pod set and grain filling stages for the Vmax RR cultivar, with 107 and 160 mm recorded at GUA1, 86 and 141 mm recorded at GUA3, and 72 and 133 mm recorded at GUA5, respectively (Table 5). The water deficit calculated during the flowering to grain set stages (R1–R5) and grain set to physiological maturity stages (R5–R7) using the indices (R-ETo) were −168 and −80 mm at GUA1, −192 and −113 mm at GUA3, and −205 and −102 at GUA5, respectively. In addition, the water stress indices showed values of 9.6 and 14.0 at GUA1 to 14 and 15.8 at GUA3 and 17 and 14.9 at GUA5 for the same phenological stages. The same water regime was observed for the CD202 cultivar. The lower rainfall at GUA3 and GUA5 relative to that at GUA1 (Table 5) was exacerbated by poorly distributed rainfall, especially during the flowering-pod set stage (Fig. 6a). Thus, significant rainfall occurred immediately after flowering, which was followed by a period without rainfall that was interrupted by 19 mm of rainfall in 2 days at GUA1; however, the dry period extended for another week at GUA3 and GUA5. Thus, reduced and poorly distributed rainfall at GUA3 in GUA5 resulted in more severe water stress during the pod set stage, which was responsible for the decreased soybean production by approximately 1.3 t at these sites compared with the GUA1 site.

**Table 4:** Observed and estimated yields and yield components according to crop season, transect site, and cultivar

Crop season	Site	Cultivar	Yield (kg ha^−1^)		Yield components			
			Observed	Estimated^a^	PM2	PP	GP	UGW (mg)
2011/2012	GUA1	Vmax	4727	4749	29.6	45.9	2.44	143
	GUA3		3392	3510	27.0	39.3	2.35	141
	GUA5		3669	3387	28.1	33.8	2.34	153
	GUA1/GUA3		1.39	1.35	1.10	1.17	1.04	1.02
	GUA1/GUA5		1.29	1.40	1.05	1.36	1.04	0.93
	GUA1	CD202	4647	4671	24.9	65.1	2.03	142
	GUA3		3397	3201	26.5	50.0	1.95	124
	GUA5		2997	2850	25.2	44.3	1.86	137
	GUA1/GUA3		1.37	1.46	0.94	1.30	1.04	1.04
	GUA1/GUA5		1.55	1.64	0.99	1.47	1.09	1.03
	SHE4	Vmax	3263	3001	31	30	2.40	135
	SHE5		2511	2301	31	23	2.33	139
	SHE4/SHE5		1.30	1.30	1.00	1,30	1.03	0.97
	SHE4	CD202	3131	3075	24	50	2.05	125
	SHE5		2262	2132	25	30	2.06	138
	SHE4/SHE5		1.38	1.44	0.96	1.67	1.00	0.91
	STI1	Vmax	2965	2901	25.9	42.7	2.16	121
	STI5		2167	2098	24.0	34.0	2.36	109
	STI1/STI5		1.37	1.38	1.08	1.26	0.92	1.11
	STI1	CD202	2651	2762	24.8	48.1	2.08	119
	STI5		1737	1763	22.4	43.1	2.09	88
	STI1/STI5		1.53	1.57	1.11	1.12	1.00	1.35
	STI1	Vmax	4041	4445	32.0	42.5	2.27	144
	STI4		3085	2934	33.7	31.1	2.24	125
2013/2014	STI1/ST4		1.31	1.52	0.95	1.37	1.01	1.15
	STI1	CD202	3823	4434	24.0	67.3	2.03	135
	STI4		3288	3272	25.3	46.1	2.08	135
	STI1/ST4		1.16	1.36	0.95	1.46	0.98	1.00
	SHE1	Vmax	3384	3500	32.7	34.9	2.31	132
	SHE2		2745	2813	33.1	30.0	2.18	130
	SHE1/SHE2		1.23	1.24	0.99	1.16	1.06	1.02
	SHE1	CD202	3287	3531	24.0	59.9	2.01	123
	SHE2		2813	2944	24.5	50.7	1.99	119
	SHE1/SHE2		1.17	1.20	0.98	1.18	1.01	1.03

*PM2* plants m^−2^, *PP* pods plant^−1^, *GP* grains pod^−1^, *UGW* unit grain weight

^a^Estimated yield = (PM2 PP GP UGW)/100

**Table 5:** Rainfall, rainfall minus the reference evapotranspiration (R-ETo), and water stress at different phenological stages (Fehr and Caviness 1977) according to crop season, transect site, and cultivar

Crop season	Site		Rainfall (mm)				R-ETo (mm)				Stress			
			V1–R1	R1–R5	R5–R7	V1–R7	V1–R1	R1–R5	R5–R7	V1–R7	V1–R1	R1–R5	R5–R7	V1–R7
2011/2012	GUA1	Vmax	129	107	160	396	−119	−168	−80	−361	0.0	9.6	14.0	22.7
	GUA3		191	86	141	418	−59	−192	−113	−358	0.1	14.0	15.8	28.9
	GUA5		206	72	133	411	−44	−205	−102	−344	0.1	17.0	14.9	31.0
	GUA1	CD202	193	64	139	396	−91	−187	−89	−361	0.0	11.2	12.4	23.1
	GUA3		251	45	122	418	−34	−210	−120	−358	0.4	15.4	14.6	29.7
	GUA5		244	52	115	411	−41	−202	−107	−344	0.7	17.9	13.7	31.5
	SHE4	Vmax	205	68	181	454	−14	−158	−40	−212	0.0	7.13	11.2	SH4
	SHE5		199	71	158	428	−41	−180	−90	−311	0.0	9.9	16.9	SH5
	SHE4	CD202	264	19	173	454	26	−221	−55	−249	12.0	21.51	12.0	SH4
	SHE5		257	20	152	428	−3	−248	−104	−352	17.3	30.34	17.3	SH5
	STI1	Vmax	252	95	61	409	39	−164	−139	−265	0.0	11.8	16.9	28.1
	STI5		151	57	59	267	−67	−208	−144	−419	0.0	15.7	18.7	33.6
	STI1	CD202	262	97	61	409	1	−162	−139	−265	0.3	16.0	17.4	28.6
	STI5		154	65	59	267	−113	−199	−144	−419	0.0	18.0	18.8	33.8
2013/2014	STI1	Vmax	214	138	104	456	−38	−93	−97	−229	0.7	0.00	6.1	6.8
	STI4		204	115	97	416	−53	−115	−105	−273	0.7	0.41	11.0	11.8
	STI1	CD202	214	138	116	468	−53	−78	−89	−220	0.7	0.02	6.5	7.2
	STI4		208	111	135	454	−63	−104	−70	−237	0.7	3.22	12.1	12.99
	SHE1	Vmax	202	85	115	402	−51	−125	−112	−287	0.0	5.6	7.1	12. 7
	SHE2		197	54	129	380	−58	−161	−103	−321	0.0	8.4	6.8	15.3
	SHE1	CD202	204	83	116	403	−88	−88	−122	−298	0.0	5.6	8.2	13.8
	SHE2		199	52	132	383	−95	−123	−111	−329	0.0	8.5	8.5	17.0

Differences among values that gradually decreased from the reservoir shoreline also occurred during the 2011/2012 crop season in STI (Table 2) because of higher yield at the site near the reservoir (STI1) compared with the remaining sites (Fig. 3 and Table 4). The decreasing yield of the Vmax RR and CD202 cultivars from sites 1 to 5 may be attributed to the reduced number of pods per plant (26 and 12%) and grain unit weight (11 to 35 %) as well as to the reduced plant stand (8 and 11 %) (Table 4). From STI1 to STI5, the rainfall decreased from 409 to 267 mm during the development cycle (V1–R7) and from 97 to 57 mm during the flowering to pod set stage (R1–R5) (Table 5). The estimated water deficit and water stress indices were inversely proportional to the reduced yield, indicating that water availability was the main determinant of the yield.

The yield decrease in SHE5 as compared to SHE4 in the 2011/12 crop season and the lower yields in STI4 and SHE2 during the 2013/2014 crop season relative to the sites closer to the reservoir in each transect can be also attributed to water stress. Lower rainfall was observed during the flowering-grain set stage (R1–R5) (Table 4 and Fig. 6b, c), which is consistent with the reduced number of pods per plant and compromised yields at these sites.

## Conclusions

Experiments conducted during 4 years, with two cultivars and considering three transects with five distances from the Itaipu reservoir, provided data for comparison between soybean yields at sites closer to the reservoir and yields from sites farther away in each transect. The results indicated that the yield varied with distance but not in a consistent way. Consequently, the Itaipu reservoir does not affect the yield of soybean plants grown within approximately 10 km from the shoreline.

The similarity of the dates of the different phenological stages among the sites in the same transect confirmed that thermal uniformity occurred along the transects.

The low correlation between air temperature and soybean yield confirmed that variations in the observed yield among the sites in the transects were not caused by the thermal conditions measured during the experimental period.

Water availability was responsible for the highest differences in yield among sites in the same transect, with water stress caused by the spatial variability in rainfall, not related to distance from the reservoir, especially during the soybean reproductive period in January and February, reducing the number of pods per plant and grain unit weight.

## Electronic supplementary material

Below is the link to the electronic supplementary material.

ESM 1 (DOCX 1943 kb)
